# Immunohistochemical distribution of Bcl-2 and p53 apoptotic markers in acetamiprid-induced nephrotoxicity

**DOI:** 10.1515/med-2022-0603

**Published:** 2022-11-21

**Authors:** Gokhan Nur, Emrah Caylak, Pinar Aksu Kilicle, Safak Sandayuk, Ozlem Onen Celebi

**Affiliations:** Department of Biomedical Engineering, Faculty of Engineering and Natural Sciences, Iskenderun Technical University, Hatay, Turkey; Department of Biochemistry, Faculty of Medicine, Girne American University, Kyrenia, Cyprus; Department of Molecular Biology, Faculty of Science and Arts, Kafkas University, Kars, Turkey; Department of Zoology, Faculty of Science and Arts, Kafkas University, Kars, Turkey

**Keywords:** kidney, acetamiprid, bcl-2, p53, histopathology, immunoreactivity

## Abstract

Pesticides, which adversely affect the critical metabolic processes of organisms, disrupt the physiological balance by specifically targeting enzymes and may lead to such consequences that may lead to death. It provides benefits in agricultural activities. The p53 protein antagonizes bcl-2, an anti-apoptotic protein character, and induces apoptosis by causing mitochondrial membrane permeability. This study aims to show the effect of acetamiprid, which is an insecticide from the neonicotinoid class, on bcl-2 and p53 immunoreactivity, which has an important place in the apoptotic mechanism in kidney tissue. A total of four groups including control and three experimental groups (the acetamiprid was administered 5, 10, and 15 mg kg^−1^) were formed in the study. After acetamiprid was administered via gavage for 14 days, the kidney tissues taken from the mice, which were sacrificed by cervical dislocation, were fixed in 10% formaldehyde solution for histological and immunohistochemical analyses, and as a result of routine tissue follow-up, the sections were blocked in paraffin and stained with haematoxylin–eosin and immunostaining. The histopathological examinations revealed that while the kidney tissue had a normal structure in the control group, degeneration in the distal and proximal tubules, glomerular degeneration, increase in the capsular area, glomerular atrophy, and haemorrhage were determined in the acetamiprid groups at increasing severity and frequency depending on the dose of the applied substance. In the kidney tissue, Bcl-2 and p53 immunoreactivity was observed in glomerular cells, sinusoidal epithelium, and proximal and distal tubule cells. The acetamiprid caused pathological changes in the kidneys in the dose range used. This effect also affects the expression of bcl-2 and p53 genes, which are biomarkers in the apoptotic mechanism. As acetamiprid accumulates in tissues, it increases the expression of p53 from cell death receptors, while suppressing the anti-apoptotic bcl-2 expression.

## 1 Introduction

Pesticides, which adversely affect the critical metabolic processes of organisms, disrupt the physiological balance by specifically targeting enzymes and may lead to such consequences that may lead to death. It provides benefits in agricultural activities; however, it is accepted as an important risk factor for the environment and humans due to its toxicity [1,2]. Insecticides, which are widely used for growing agricultural products and preserving the crop, are synthetic pesticides that are used in insect control, especially against infectious diseases [3]. Organophosphates, which are widely used in insecticides, have toxic effects. It acts by increasing the amount and duration of action of the neurotransmitter by blocking the normal breakdown of acetylcholine [4]. Neonicotinoids, a widely used class, have started to be used more than organophosphates in recent years [5]. They are present at the synaptic junction as acetylcholine agonists and bind to the acetylcholine receptors of the central nervous system in insects, leading to stimulation by interrupting synaptic transmission [6]. Neonicotinoids are less resistant, more effective, and less toxic to organisms other than the target organism compared to other pesticides [5]. One of the active substances of these drugs, acetamiprid [*N*-(6-Chloro-3-pyridylmethyl)-*N*-cyano-*N*-methylacetamidine], is the most commonly produced neonicotinoid class insecticide after imidaclopride. Except for the purpose for which it is used in nature, it tends to accumulate in kidney, liver, adrenal, and thyroid gland tissues in other living beings, and 50–70% of them is excreted as metabolites via urine and faeces. It is explicit that the residues of this insecticide, used in agricultural activities, may contaminate the environment and nutritional sources and, thus, may act as a potential agent that may disrupt human health as a result of accumulation in human organisms, and the study data for this issue have not been at the desired level, yet [3,7,8].

Apoptosis is a natural mechanism that removes the undesired cells from the body and plays an important role in organism development and tissue homeostasis [9]. The irregularity of the mitochondrial pathway and resistance to apoptosis, which are the characteristics of cancer, are controlled by B cell lymphoma-2 (bcl-2) members. The tumour suppressor protein p53 may mediate apoptosis via transcriptional regulation of some pro-apoptotic genes, such as the death receptor [10]. The activation of mitochondria is important in the irreversible apoptotic process. The bcl-2 family can be regarded as the most important factor causing this. This family fulfils the reverse functions as anti-apoptotic (bcl-2, bcl-XL) and pro-apoptotic (Bax, Bad) [11]. In the apoptotic process, bcl-2, which is an anti-apoptotic member of the BCL-2 family, functionally inhibits apoptosis by directly binding BAK and BAX effector proteins or only pro-apoptotic BH3 members. Studies are carried out especially on bcl-2, Bax, and caspases in the investigation of apoptosis-related pathways [12,13,14].

Tis study aims to reveal the apoptotic potential of acetamiprid, one of the neonicotinoid group insecticides, through its effects on bcl-2 and p53 expression and the changes it causes in kidney tissue in order to determine its effects on organisms.

## 2 Materials and methods

### 2.1 Chemical

Anti-bcl-2 antibody, anti-p53 antibody, 3-amino-9-ethylcarbazole, Histostain-Plus Bulk Kit chemicals, and water-based adhesive used in the study were supplied from Abcam, Invitrogen, Thermo Fisher Scientific, and Merck companies. Achieve branded 20% acetamiprid (Sefa agriculture registry No: 5869) was used as acetamiprid. All the other chemicals and reagents used in the study were selected by considering the criteria of being high-purified.

### 2.2 Animals and treatment

In this study, 40 *Mus musculus* var. *Albinos* male mice which were approximately 8 weeks old and weighing 25–30 g were used in the study. The mice were fed ad libitum with normal mouse feed and tap water. The mice were kept in cages made of autoclavable polycarbonate material at 121°C under ambient conditions including a temperature of 21 ± 2°C, the humidity of 50%, and 12 h light and 12 h dark cycle. The dose of the substances to be administered was determined according to the daily weight of the animals. After these substances were dissolved in distilled water, they were administered to the mice by oral gavage. Forty male mice used in the study were randomly divided in four equal groups according to the application protocol below. At the end of the study protocol, the animals were sacrificed via the cervical dislocation method under ketamine/xylazine anaesthesia. Then, the kidneys resected by making a vertical incision from the periumbilical region were weighed on an analytical balance and then normalized with live weight and used in the statistical analysis.

Group 1. (Negative Control Group, *n:* 10): Distilled water was administered to the mice in the negative control group via oral gavage.

Group 2. (5 mg kg^−1^ Acetamiprid group, *n*: 10): 5 mg kg^−1^ acetamiprid was administered to the mice in this group via oral gavage for 14 days.

Group 3. (10 mg kg^−1^ Acetamiprid group, *n*: 10): 10 mg kg^−1^ acetamiprid was administered to the mice in this group via oral gavage for 14 days.

Group 4. (15 mg kg^−1^ Acetamiprid group, *n*: 10): 15 mg kg^−1^ acetamiprid was administered to the mice in this group via oral gavage for 14 days.

### 2.3 Organosomatic index

The weights of the kidneys were recorded, and the following formula was used to calculate the organosomatic index:

\text{Organosomatic}\hspace{.25em}\text{index}=(\text{Tissue}\hspace{.25em}\text{weight}/\text{Body}\hspace{.25em}\text{weight})\hspace{1em}\times 100.]

### 2.4 Histological analysis

The kidney tissue taken from mice, which were sacrificed by cervical dislocation, was fixed in a 10% buffered formaldehyde solution in order to be used for histological and immunohistochemical analyses. These fixed tissues were routinely blocked in paraffin by passing through graded alcohol series, methyl benzoate, and benzole, and 5 µm serial sections were taken from the blocks on slides coated with chrome alum gelatin. Tissue sections on slides were stained with haematoxylin & eosin [15]. At the end of the staining procedure, one drop of entellan was dropped on the tissue section and then covered with lamella. Preparations were examined under a light microscope (Zeiss Primo Star). The intensity of the histopathological changes was provided in a semi-quantitative manner by using the grading system of Sakat et al. [16].

### 2.5 Immunohistochemical analysis

In order to determine the immunoreactivity of Bcl-2 and p53 in the kidney tissues of mice, these tissues were fixed in a 10% formaldehyde solution and then blocked in paraffin by passing through graded alcohols, methyl benzoate, and benzoles. Anti-Bcl-2 (B-cell lymphoma 2, ab194583) and anti-p53 (tumour protein, ab131442) primary antibody was diluted at 1/100 and applied in a humid environment for 1 h at room temperature to examine the immunoreactivity of Bcl-2 and p53 on 5 μm sections taken from paraffin blocks. Meanwhile, only Phosphate buffer solution was dropped on the tissues for the negative control group. After the incubation of primary antibodies, the Streptavidin-biotin peroxidase technique, one of the indirect methods, was used [17]. HRP streptavidin (Invitrogen Histostain plus Broad Spectrum –AEC, Ref. 85.9943) was applied to the sections, and then, they were incubated at room temperature for 15 min. AEC (3-amino-9-ethylcarbazole) solution was added to the sections for chromogen application. Then, the sections were examined under a light microscope, immunoreactivity control was performed, and the reaction was deactivated with distilled water according to the immunoreactivity status. The sections were then immersed in haematoxylin for negative staining and coated with a lamella by using a water-based adhesive (Lab Vision, Large Volume Vision Mount, TA-060-UG). Considering random area selection, the photographs were taken by two researchers under a light microscope, and the sections were graded as none (0), mild (1), moderate (2), and severe (3) according to their immunoreactivities [18,19]. Accordingly, if there is no positive cell, they are rated as 0; if the rate of positively stained cells is <1/100, they are rated as 1; if the rate of positively stained cells is between 1/100 and 1/10, they are rated as 2; if the rate of positively stained cells is between 1/10 and 1/3, they are rated as 3; if the rate of the positively stained cells is between 1/3 and 2/3, they are rated as 4; if the rate of positively stained cells is >2/3, they are rated as 5. Then, a score expressing the mean intensity of positive cells was calculated. Accordingly, it was evaluated as 0 in case of no staining, 1+ in weak immunoreactivity, 2+ in moderate immunoreactivity, and 3+ in strong (increased) staining.

### 2.6 Statistical analysis

Statistical Package for Social Sciences 22.0 software was employed for statistical evaluation. The results were statistically analysed using one-way analysis of variance (one-way ANOVA). Statistical significance between the groups was assessed using Tukey HSD multiple comparison test. The threshold value of significance was accepted as *p* < 0.05. Results were represented as mean ± standard deviation.


**Ethical approval:** This study was approved by Kafkas University Animal Experiments Local Ethics Committee (permission no. 17.03.2017/036).

## 3 Results

### 3.1 Kidney weight findings

Kidneys taken from the animals which were sacrificed via cervical dislocation after anaesthesia were weighed and normalized with live weight [(kidney weight/live weight) × 1,000]. Table 1 shows the changes caused by acetamiprid in the kidney organ weight. When the data were evaluated, it was observed that acetamiprid exposure caused an increase in the kidney tissue. When compared with the control group, it was determined that the application of the lowest dose of acetamiprid did not cause a statistically significant increase in the weight of the kidney tissue. A statistically significant increase in the kidney weight than the control group was observed in 10 mg kg^−1^ acetamiprid group. Weight gain in the 15 mg kg^−1^ group, which is the highest application dose of acetamiprid, was very limited compared to the 10 mg kg^−1^ acetamiprid group, and there was no statistically significant difference between 15 mg kg^−1^ acetamiprid group and 10 mg kg^−1^ acetamiprid group. After the increase in the kidney tissue was evaluated between the groups, the highest increase was observed in the 15 mg kg^−1^ acetamiprid group and increased by 35.29% than in the control group.

**Table 1:** Effects of acetamiprid on the kidney organ weight (g) between the groups

Parameter	Groups				*p*
	Control (*n*: 10)	5 mg kg^−1^ Acetamiprid (mean ± SD) (*n*: 10)	10 mg kg^−1^ Acetamiprid (mean ± SD) (*n*: 10)	15 mg kg^−1^ Acetamiprid (mean ± SD) (*n*: 10)	
(Kidney weight/live weight) × 1,000 (g)	0.34 ± 0.06^c^	0.36 ± 0.06^b,c^	0.44 ± 0.06^a,b^	0.46 ± 0.07^a^	*

^*^ *p* < 0.01, Statistically significant difference; ^a,b,c^, values with different superscript letters indicate significant differences; *n*, number of animals in the group; SD, standard deviation.

### 3.2 Histopathological findings

**Figure 1:**
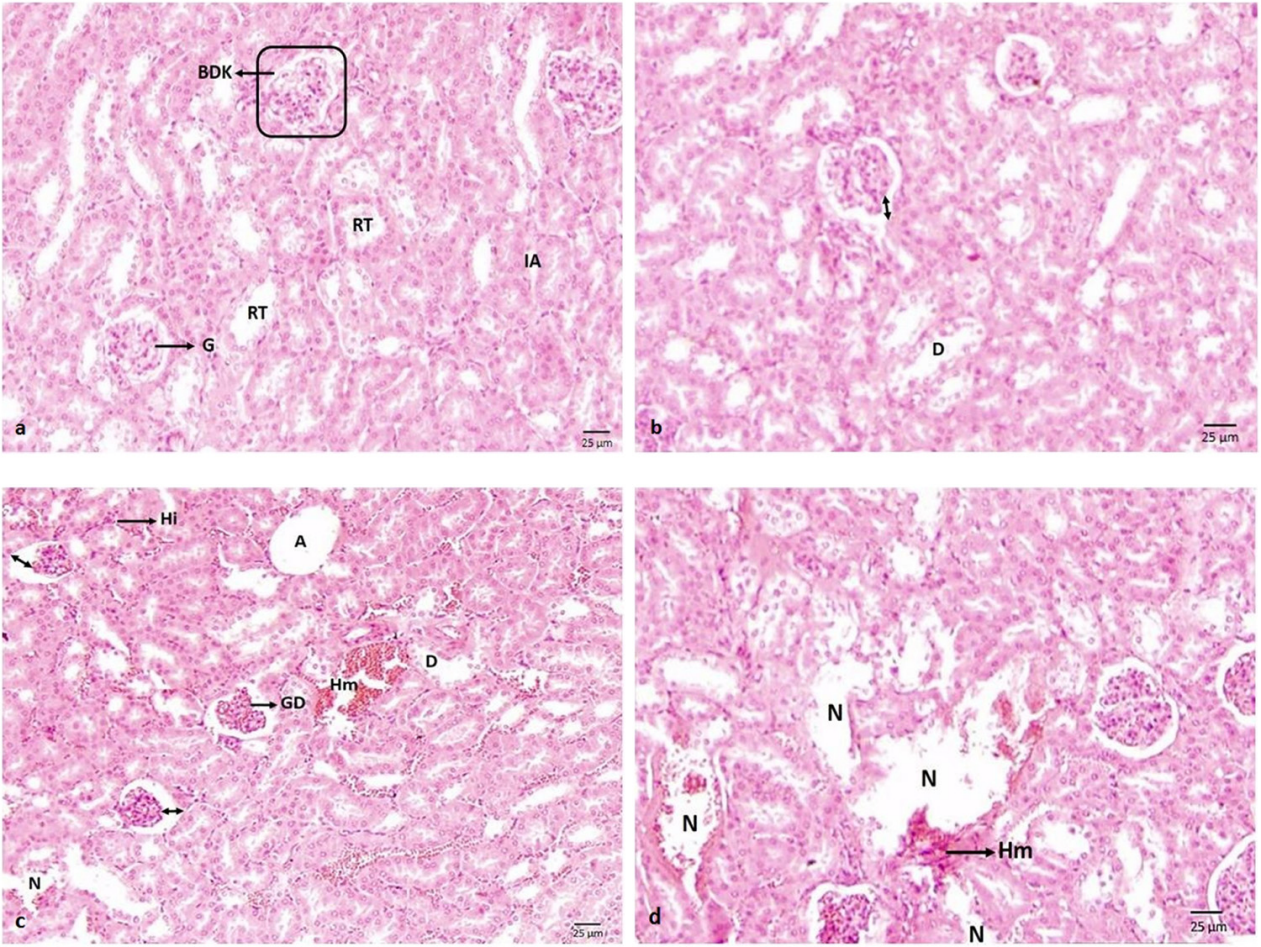
(a) Kidney histology of negative control group; (b) histological structure of the kidney in the 5 mg kg^−1^ acetamiprid group; (c) histological structure of the kidney in the 10 mg kg^−1^ acetamiprid group; and (d) histological structure of the kidney in the 15 mg kg^−1^ acetamiprid group, RT, IA, glomerulus (G), malpighian corpuscle (rounded rectangle), CTC, degeneration (D), increase in capsular area (bowman) (↔), GD, hyperchromasia (Hi), haemorrhage (Hm), atrophy (A), necrosis (N), and Bar: 25 µm, H&E.

As seen in the samples of the control group, the kidney structure consists of renal tubules (RT) that are embedded in the parenchyma consisting of interrenal cells. It was observed that erythropoietic and granulopoietic series, lymphocytes, and phagocytes were present in the interrenal area (IA). RT are distinguished by their capsules surrounding their smooth circular and ellipsoid structures, and light-coloured epithelial cells with large nuclei and well-defined borders. The glomerulus, in the form of a capillary glomerulus, forms the malpighian corpuscle that is externally surrounded by a connective tissue capsule (CTC). IA is quite narrow. Even though a mild degeneration was observed in the distal tubules and the capsular area increased in the kidney histology in the group of 5 mg kg^−1^ acetamiprid, the general appearance was similar to the control group. In the 10 mg kg^−1^ acetamiprid group, it was observed that degeneration in the distal tubules increased more and reached necrotic sizes in the kidney histology. Glomerular atrophy was observed from place to place in addition to the glomerular degeneration (GD) occurring in the form of changes in the glomerulus morphology. Upon the increase in the capsular area, the bowman’s capsule disappeared as a result of the adhesion of glomerulus to the parietal leaves of the bowman’s capsule. Thus, it was found that glomerulus almost completely filled the bowman’s capsule, and an appearance with no bowman’s capsule was observed. Haemorrhage was in question in addition to the significant hyperchromatic appearance of the IA. In the general appearance of kidney histology, haemorrhage and necrosis in the tubules reached the maximum dimensions in the 15 mg kg^−1^ acetamiprid group (Figure 1a–d). Table 2 shows the adapted tissue change frequency ratings of histopathological lesions in the kidney.

**Table 2:** Tissue change ratings of the histopathological lesions in the kidney tissue in *Mus musculus* var. *albinos* (frequency ratings were adapted from Sakat et al. [16])

Tissues	Lesions		Acetamiprid dose groups		
		Control group	5 mg kg^−1^	10 mg kg^−1^	15 mg kg^−1^
Kidney	GD	0	0	+	++
	Hyperchromasia	0	0	+	++
	Glomerular atrophy	0	+	++	+++
	Degeneration in tubular epithelium	0	+	++	+++
	Hematopoietic tissue loss and necrosis	0	0	+	+++
	Haemorrhage	+	+	++	++

0, No abnormality; +, abnormality frequency is low; ++, abnormality frequency is moderate; and +++, abnormality frequency is high.

### 3.3 Immunohistochemical findings

**Figure 2:**
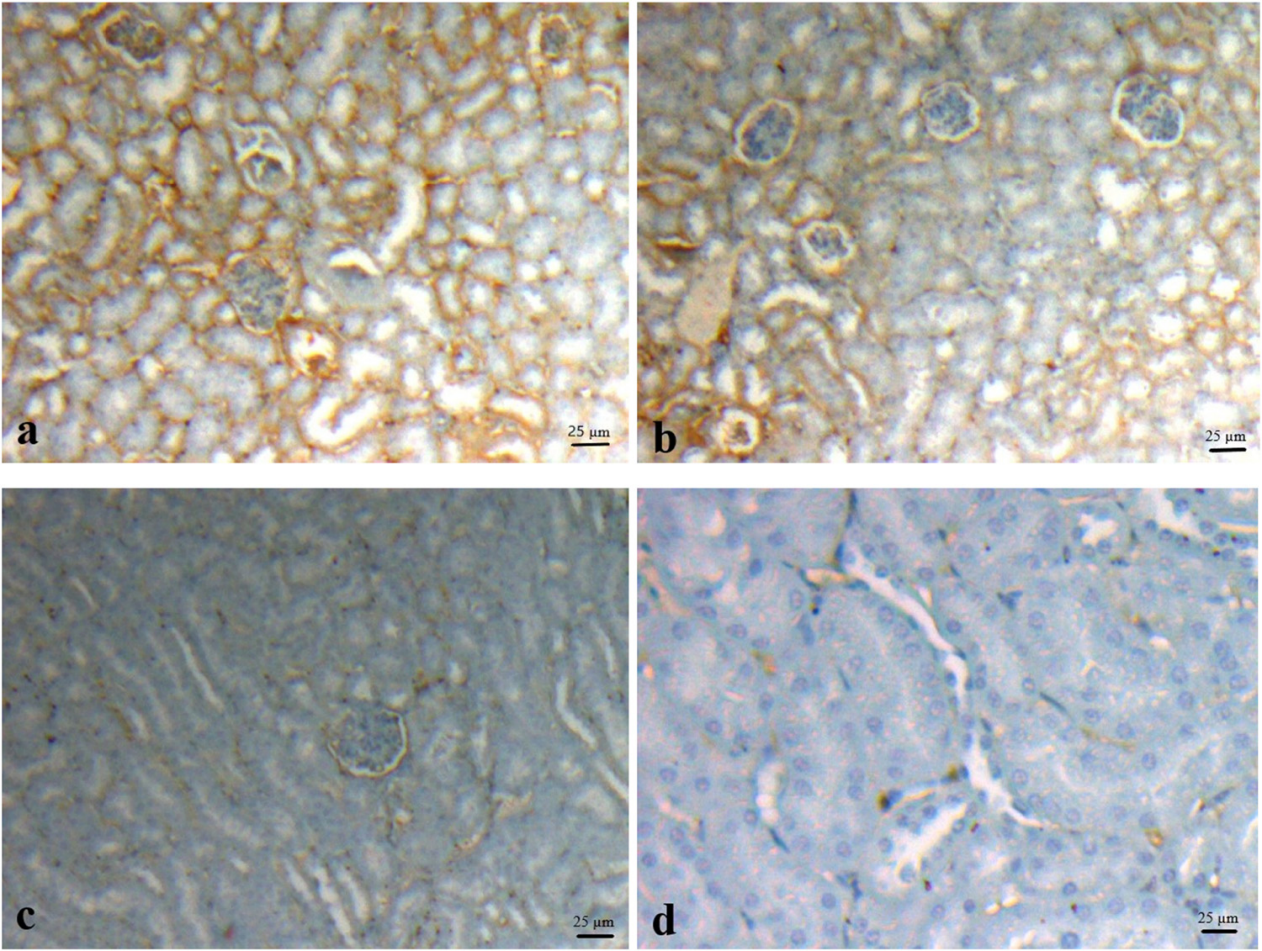
In the kidney tissue, bcl-2 immunoreactivity was observed in the tubule cells, sinusoidal epithelium, and glomerular cells in all the groups. (a) Dense immunoreactivity in the cortex region of the kidney tissue obtained from the control group. (b) Moderate immunoreactivity in the kidney cortex area in 5 mg kg^−1^ acetamiprid group. (c) Poor immunoreactivity in the kidney cortex–medulla area in 10 mg kg^−1^ acetamiprid group. (d) Very poor immunoreactivity in the kidney cortex–medulla area in 15 mg kg^−1^ acetamiprid group, IHC-P, bar: 25 µm.

**Figure 3:**
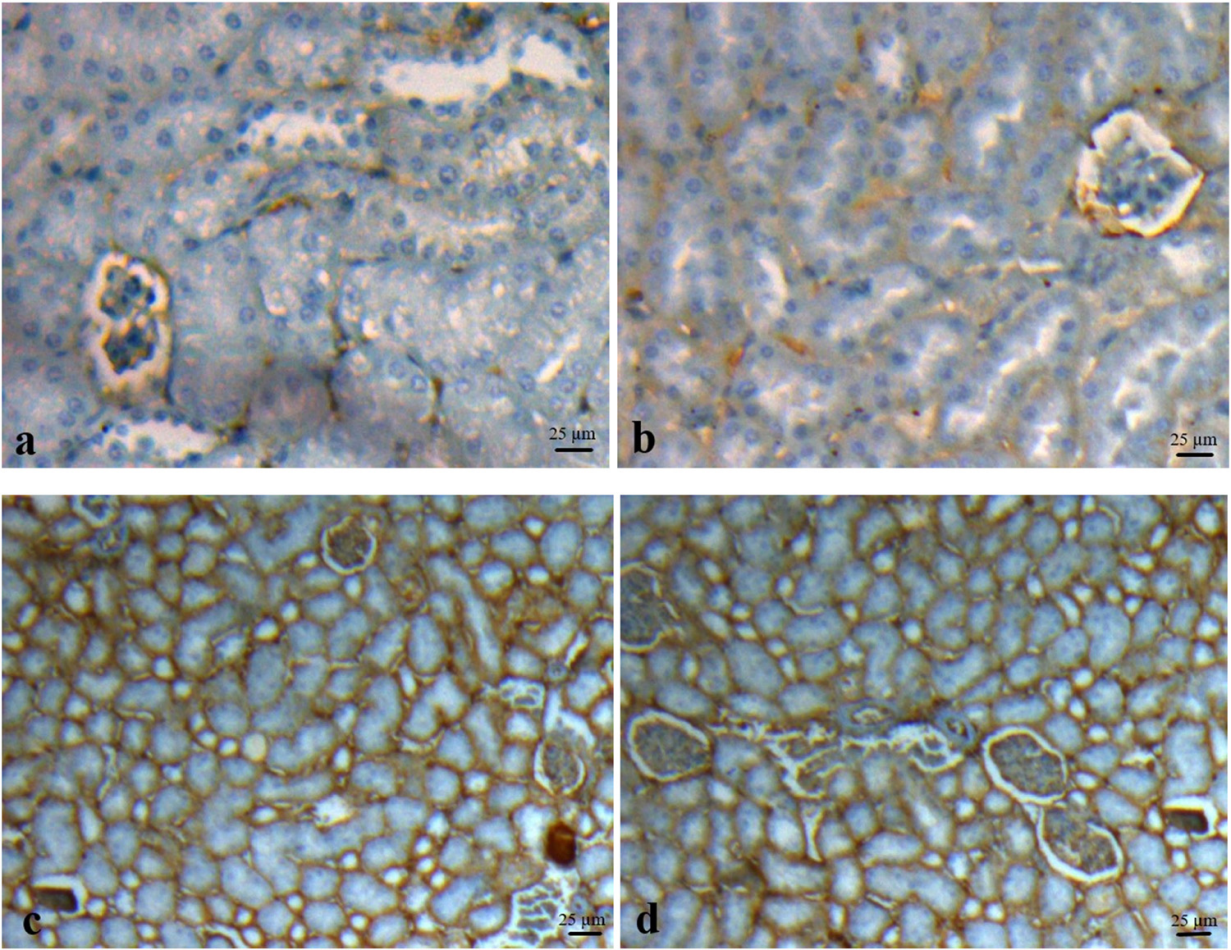
p53 immunoreactivity in the kidney tissue: (a) Quite poor immunoreactivity in the control group, (b) moderate immunoreactivity in the sinusoidal epithelium in 5 mg kg^−1^ acetamiprid group and poor immunoreactivity in the tubular epithelium and glomerular cells, (c) strong immunoreactivity in the sinusoidal epithelium and glomerular cells in 10 mg kg^−1^ acetamiprid group, and (d) strong immunoreactivity in the sinusoidal epithelium and glomerular cells in 15 mg kg^−1^ acetamiprid group, and moderate immunoreactivity in the tubular epithelium, IHC-P, bar: 25 µm.

As a result of the immunohistochemical evaluation, kidney bcl-2 and p53 immunoreactivity was observed in glomerular, sinusoidal epithelium, distal, and proximal tubule cells. In the kidney tissue obtained from the control groups, bcl-2 expression showed denser immunoreactivity than the acetamiprid groups (Figure 2a–d). In the kidney tissue obtained from the acetamiprid groups, p53 reactivity was denser than in other groups (Figure 3a–d). It was determined that bcl-2 and p53 immunoreactivity was higher in the cortex region of the kidney than in the medulla part.

While p53 immunoreactivity was strong in the glomerular cells and sinusoidal epithelium in the 10 and 15 mg kg^−1^ acetamiprid groups, it was observed to be less dense in the tubular epithelium. The immunoreactivity, which was observed at moderate density in the 5 mg kg^−1^ acetamiprid group, was detected as poorer in the control group.

## 4 Discussion

In this study, it was determined that acetamiprid, a neonicotinoid class insecticide, has caused the pathological phenomena in the kidneys at the doses used, increased the expression of p53, a cell death receptor from apoptotic biomarkers, due to dose accumulation in tissues, and reduced the expression of anti-apoptotic bcl-2 due to tissue destruction. Pesticides have been used for veterinary medicine and public health apart from agricultural activities against various pests. As a result of the use of pesticides, it has been observed that the yield in plant production is protected and increased, and the effects of diseases are reduced, while the unconscious and long-term use of pesticides is regarded as a contaminant as a result of their accumulation in agricultural lands [20,21], food [22] and water [23]. Neonicotinoids have become the most commonly used class of insecticides throughout the world. Although numerous studies have documented neonicotinoid toxicity in bees and other insects, the effects of exposure on various tissues in mammals are not substantially investigated [24]. The toxicity of neonicotinoids in mammals is similar to the symptoms emerging as a result of nicotine poisoning [25]. Although it is accepted as safe in humans compared to other classes of insecticides, recent studies have indicated that it has the potential to cause a disorder in the human metabolism [26,27,28]. Acetamiprid, belonging to the neonicotinoid class, is a broad-spectrum insecticide used for the protection of vegetables and fruits from insects.

In the present study, it was found that acetamiprid exposure caused an increase in the weight of kidney tissue. Among the studies of other researchers using organ weight, a study in which cypermethrin application was made, reported that while the liver tissue decreased in volume and weight, weight increased in the kidney tissue [29]. In another study, it was found that fipronil caused an increase in liver and kidney weights [30]. Conversely, some insecticides cause a decrease in the body and organ weights. Methyl parathion caused a decrease (by weight) in the body weight and kidney tissue [31]. In a study in which malathion was applied, it was reported that kidney weight decreased compared to the control group [32]. As in the present study, as a result of pesticide applications, enzymatic activity increases due to overwork in the kidneys, and ultimately kidney weight, and volume increase.

In a study that examined the toxic effect of acetamiprid in mice, atrophy of glomerulus, enlarged capsular spaces, and swelling, vacuolization, and degeneration of proximal and distal tubule epithelium were observed in kidney tissue. In renal failure, especially caused by acetamiprid, the structure of renal corpuscles and tubules gets damaged by strengthening the role of nitric oxide in such a failure. Lower acetamiprid concentration in the kidneys than in the liver suggests that its harmful effects on the kidney may be caused by the acetamiprid metabolites. Finally, after an acetamiprid exposure, findings of an increase in the kidney tissue and capsular area, GD, hyperchromasia in the IA, haemorrhage, tubular degeneration, atrophy, and necrosis were observed, and the severity of the findings increased in parallel with the dose increase [33]. Since the kidneys perform the tasks of protecting osmoregulation and ion balance in general, these findings are thought to be encountered as a result of disruptions in the intrarenal water or ion balance [34]. As a result of the application of pesticides with similar properties, similar structural changes are observed in the tissues. Upon Dichlorvos application, enlargement in the bowman’s capsule in the kidney tissue, inflammatory cell infiltration, glomerular atrophy, vascular obstruction, and tubular degeneration were stated [2]. After malathion application, separation from the basal membrane, mononuclear cell infiltration, and glomerular atrophy in the kidney tissue were observed [32]. In another study, it was reported that morphological and pathophysiological changes have been observed due to the formation of reactive oxygen species as a result of chlorfenvinphos application [35]. Similar toxicity findings have been also reported by different researchers before [36,37,38]. The findings obtained as a result of the present study show parallelism with the nephrotoxicity of acetamiprid and similar pesticides used in other studies on the kidneys.

Apoptosis stimulation is quite important for the tumour suppressing activity of p53 [39]. p53, a nuclear transcription factor, regulates numerous genes in cell apoptosis. Especially, it is understood that it mediates the mitochondrial apoptosis pathway, also called the intrinsic pathway, where p53 migrates to the mitochondria. Tumour suppressing feature of the p53 protein regulates the cell cycle by preventing the rapid and uncontrolled growth and division of the cells [40,41]. Proteins in the BCL-2 family, containing both pro-apoptotic and anti-apoptotic members, control the mitochondrial outer membrane permeability and thus the activation of the caspase cascade [42]. The mixtures of various pesticides with each other can reveal different results from their sole uses. In addition, the effects of each pesticide on the expression of apoptotic markers are different. In a study that used only acetamiprid, Caspase 8 (Cas8) mRNA expression level showed a significant increase than the control group. However, no significant difference was observed at the expression level when only iprodione, pyrimethanil, or pyraclostrobin was used. Caspase 9 (Cas9) mRNA levels significantly decreased in the group where only iprodione or iprodione + pyrimethanil were used together when compared to the control group. The p53 mRNA levels decreased significantly in all groups where the applied pesticides were used individually or in combination. In addition, Bcl-2-related X protein (Bax) mRNA levels were determined as significantly downregulated in the single or double mixture groups [43]. Depending on the toxic effect caused by lambda cyhalothrin, one of the pesticides in a similar group, p53 mRNA and protein expression values increased and Bcl-2 mRNA and protein expression levels decreased. On the other hand, it was reported that p53 expression decreased and Bcl-2 mRNA and protein expression levels increased as a result of the application of Panax ginseng, with a known antioxidant effect, together with lambda-cyhalothrin [44]. There are numerous factors that are effective on p53 and Bcl-2 expression and kidney weight in the kidney tissue. While p53 expression increased in kidney glomerulus and medullary tubules in the rats with polycystic ovary syndrome, anti-apoptotic Bcl-2 expression decreased in kidney glomerulus, and increased p53 and decreased Bcl-2 were correlated with one another [45]. P53 expression shows an increase in renal injuries associated with chronic disorders. There is a high number of studies investigating the effects of some compounds at the level of p53 and bcl-2 gene expression in living organisms with the induced experimental disease model. In the experimental diabetes model, it was observed that the damage in the renal tissue was higher than that in the control group. When compared to the normal kidney tissue, p53 expression significantly increased in the group with tissue damage [46]. As a result of the acetamiprid applied at increasing doses, apoptosis, endoplasmic reticulum stress, and increase in the relevant gene expression and calcium release were reported. It was stated that there was a significant increase in bcl-2 and p53 expression as a result of exposure to increasing acetamiprid doses [47].

Neonicotinoids, which are widely used among the pesticides, and acetamiprid, a member of neonicotinoids, shows immunotoxic, nephrotoxic, neurotoxic, and hepatotoxic characteristics [44,47]. As a result of the acetamiprid application, stress on the cells occurs due to the increase in the reactive oxygen types. In this study, in which the expression levels of apoptotic markers were controlled, as a result of the induction of apoptosis depending on the increasing dose of acetamiprid application, the expression levels of p53 with the death signals produced by the cells increased; but the expression levels of Bcl-2 decreased. In the light of these results, acetamiprid is accepted as a safe option in the fight against insects; however, its use should be controlled in order to reduce its harm to mammals and to protect kidney abilities, especially since its toxic character on the kidney was revealed in this study.
